# Annual Meeting of the New York State Medical Society

**Published:** 1860-03

**Authors:** 


### PROCEEDINGS OF SOCIETIES.

### *Annual Meeting of the New York State Medical Society.*

The Annual Meeting of the New York State Medical Society convened in the Common Council Chamber, at the City Hall, Albany, February 7, at 11 o'clock, A. M.

The Society was called to order by the President, B. Fordyce Barker, who addressed the members.

Delegates in attendance then presented their credentials, which were referred to the Committee on Credentials, consisting of Drs. Percy, Willard and Hoff.

Dr. Brinsmade offered the following resolution, which was adopted:

*Resolved*, That hereafter all physicians who may be invited to take part in the deliberations of the Society must be proposed by the Committee on Credentials.

The President announced the following Committee on Nominations:

1st District—Dr. E. Harris; 2d, Dr. Wm. Govan; 3d, Dr. Mason F. Cogswell; 4th, Dr. Samuel Shumway; 5th, Dr. Wm. Taylor; 6th, Dr. Geo. W. Bradford; 7th, Dr. —— Cook; 8th, Dr. —— Mack.

Dr. Taylor offered a resolution that a copy of the Transactions of the Society be sent by the Secretary to each member of the Society. Adopted.

Dr. Blatchford, from the committee appointed at the last Annual Meeting of the Society, on the subject of the "Second Degree of Medicine," presented quite a lengthy report, and after reporting progress, asked to be continued, which was granted.

Dr. C. B. Coventry, from the committee appointed at the last Annual Meeting, under a resolution "that it is expedient to establish a Commission of Lunacy," and to report at this meeting the best method of effecting the object, presented their report and offered the following resolutions:

*Resolved*, That a petition from the Society, signed by the President and Secretary, be presented to the Legislature, in favor of the appointment of a Commission of Lunacy, in accordance with the former resolution of the Society.

*Resolved*, That a committee of ——— be appointed to confer with the committee of the Senate on the subject of such appointment.

*Resolved*, That the members of the State Medical Society, so far as practicable, confer with the members of the Legislature from their respective districts, and urge the necessity of the measure to their favorable consideration.

The report was accepted, and the resolutions set down for discussion on Wednesday morning.

Dr. Brinsmade offered the following preamble and resolution:

*Whereas*, The American Medical Association, at its last Annual Meeting, has recommended several subjects of importance to the consideration of State and County Medical Societies: Therefore,

*Resolved*, That a committee of three be appointed to examine these resolutions of the central association, and report to this Society, as early as convenient, what action, if any, may be necessary.

Adopted; and Drs. Brinsmade, Van Dyck, and Wm. Taylor were appointed such committee.

The Secretary announced a communication from the American Medical Association, on the subject of "Criminal Abortion," which was referred to the last-named committee.

A motion was made to permit Dr. Bly, of Rochester, to exhibit an artificial leg.

Objections were offered to taking up the time of the Society, thus early, in this manner, and permission was refused.

Drs. Sanders, Coates, and Bissell were appointed a committee to invite the Governor and the medical members of the Legislature to attend the sessions of the Society.

Dr. Sturtevant then read a paper on "Hypodermic Medication," which was received and referred to the Publication Committee, and the thanks of the Society were returned to the author.

An invitation was received from Gov. Morgan, inviting the officers and members of the Society to visit the Executive Mansion on Wednesday evening. Accepted.

Dr. F. J. D'Avignon read a paper on "Fracture of the lower third of the femur; two years afterwards non-union of the bones," &c. Referred to Publication Committee.

### AFTERNOON SESSION.

The Society reconvened at 3.30 p. m.

A communication was received from the Medical Society of Chenango County; also, one from the Madison County Society; also, one from the Chemung County Society. Referred to the Publication Committee.

Dr. S. D. Willard presented a biographical notice of Joel Edwin Hawley, M.D. Same reference.

The Secretary presented an obituary notice of Jonathan Purdy, M.D., by Dr. Geo. Burr; also a biographical memoir of Silas West, M.D., by Dr. Geo. Burr. Same reference.

The report of the Committee on Medical and Surgical Statistics was read by the Secretary, accepted, and the committee continued.

The Secretary read a letter from Dr. J. H. Griscom, announcing that the Common Council of the City of New York had kindly presented to him, for distribution among the members of the Society in attendance, one hundred copies of the "Report of the Proceedings and Debates of the 3d National Quarantine and Sanitary Convention."

The thanks of the Society were voted to Dr. Griscom and the authorities of New York.

Dr. S. R. Percy presented "The Transactions of the New York Academy of Medicine," vol. 2, part 4th, containing the report of the Committee on City Milk.

Dr. Finnell moved that it be referred to the Publication Committee. Adopted.

The Secretary presented the Transactions of the Medical Societies of the States of New Jersey, New Hampshire, Tennessee and Connecticut, for the year 1859.

Dr. John Ball presented a paper on "The Extirpation of the Eye." Referred to the Publication Committee.

Dr. Armstrong presented a memoir of Dr. Backus, which was referred to the same committee.

Dr. E. W. Armstrong read a paper on "Observations on Medical Prosecutions." Referred.

Dr. Mason read a communication from the Kings County Medical Society, being a history of measures adopted by it for the increase and diffusion of medical knowledge amongst its members, and of its attempts to add to the general fund of professional information. Referred to Publication Committee.

The Secretary presented from Dr. Silas Durkee his Treatise on Gonorrhœa and Syphilis, dedicated to Dr. Thomas C. Brinsmade, late President of the Society. Vote of thanks returned.

Dr. Corlies read an interesting paper on "Tumors." Referred to Publication Committee.

Dr. S. D. Willard read a paper entitled "Gun-Shot Wound within the Cavity of the Thorax." Same reference.

Dr. Charles Barrows read a paper descriptive of "A Case of Direct Inguinal Hernia." Same reference.

Adjourned until Wednesday morning, at 10 o'clock.

SECOND DAY.

The Society met at 10 o'clock yesterday morning.

The minutes were read and approved.

Dr. Griscom said that he rose for the double purpose of correcting the minutes and retrieving the injustice done to a worthy member of the Society. He alluded to the action of the Society in adopting a resolution striking from the minutes all record of a paper read by Dr. Benjamin Lee. It was stated that the gentleman was interested in the article which he had brought before the Society, but he was assured this was erroneous, and it was doing great injustice to Dr. Lee to treat his paper in this manner. He therefore moved that the minutes be amended so as to read, "The reading of the paper was suspended, and the paper referred to the Publication Committee."

Dr. McNulty objected to the motion of Dr. Griscom, and contended that it was not in order, unless a motion to reconsider was first made.

The Secretary read an explanatory letter from Dr. Lee.

Dr. Mason remarked that he had intended to bring the subject before the Society, as he was convinced injustice had been done Dr. Lee. At the proper time he conceived that it would be just to refer the paper to the Publication Committee. This could be done without amending the minutes.

The Chair decided that the motion of Dr. Griscom was in order.

Dr. B. P. Staats appealed from the decision of the Chair.

The Society sustained the Chair by a decided vote.

The question then recurred upon Dr. Griscom's motion to amend the minutes, and it was adopted, and the minutes were so amended.

The resolutions relative to establishing a Commission of Lunacy (as published yesterday) were then read.

Dr. B. P. Staats said that last year he had voted for the appointment of the committee who reported these resolutions, but he now had serious doubts as to the policy of the movement. We had had Inspectors and Commissioners for various purposes, but they had failed to satisfy the public. As the law now stands, it provides for an examination by two respectable physicians, and on their certificate that the patient is in a state of mind to render him unfit to be at liberty, he is committed by magistrates to the County Poor-House or Insane Hospital. Those physicians, generally speaking, know the patient, and are qualified to pass upon his condition. This was a fair and summary manner of disposing of him. He did not believe he would be safer in the hands of one Commissioner than under the present arrangement. More than one-half the lunatics are paupers. The expense of the Commission, besides the delay it would occasion in passing upon cases of lunacy, would be very great. He concluded by saying that he did not believe officers of this kind were needed.

Dr. Coates said that Dr. Staats's remarks were very appropriate, and if that was all the resolutions contemplated, they might better not have been originated. The examination of cases as alluded to by Dr. Staats would be but a small portion of the business devolving on this Commission. The drawer of the resolutions had in his mind views more extensive, and if the Commission should be appointed, such cases as alluded to by Dr. Staats would be left where they are now. But surrounded by laws as we are, that govern matters without, and laws within that govern mind, it seems to be necessary to legalize and systematize the matter, so that the unfortunate claimants for care can be reached and looked after.

It may be the duty of this Commission to examine alms-houses, and look up this class of beings, and have them cared for in a different manner than they now are. They are gathered together in every poor-house, and very little is done for them. This Commission *could* do something for them. But there would be a branch of business for this Commission still different. It would be the examination of insane criminals. The Governor had, last year, been called upon to examine cases of this character, and was compelled to appoint a special commission to inquire into the alleged lunacy of certain persons charged with crimes. These Commissions had disposed of the matter in a different way than a regular legalized Commission would have done.

Dr. Coates then alluded particularly to the causes above referred to. It seemed necessary to systematize this matter, and he did not see how it could be done without legislation. If this thing is to be carried out, there are some points proper to be discussed here, but for the present he would defer further remarks.

Dr. Bissell favored the adoption of the resolutions. This Commission would be appointed more particularly to inquire into the condition of lunatics in poor-houses and prisons, and ascertain who need the care and protection of the State. This is what the resolutions contemplate.

Dr. Sturtevant said he had been engaged for the past ten years in the Oneida Alms-House, and could appreciate the value of a Commission of Lunacy. He was strongly in favor of the adoption of these resolutions.

Dr. Sanders concurred with Dr. Staats as far as he went, but took a wider range. He was in favor of the resolutions, and, in case the Commission was appointed, hoped it would look into our lunatic asylums.

Dr. Coventry said Dr. Coates had presented some of the considerations that induced the presentation of these resolutions. Having been placed on the committee appointed at the last annual meeting to consider this subject, he felt bound to make a report, and after consultation with citizens in different sections of the State, both medical and otherwise, he was satisfied that something should be done for the unfortunate class of whom the Commission would have the care. But a small portion of the insane of the State find accommodation in the asylums—probably not one-half. Large numbers were congregated in the alms-houses throughout the State, and he asked where is the protection offered them by the State in these cases? Dr. Staats had entirely mistaken the objects of this proposed Commission. Its object, as set forth in the report, is, in the first place, to examine into the condition of the insane persons, wherever found in the State; and in the second place, and more particularly, to ascertain the condition of the insane, and report to the Legislature whether they are kindly taken care of. In England, they were finally driven to the necessity of applying for the appointment of a commission, and it worked admirably.

In addition to what has already been said of the duties of the Commission, they would have other and important labors to perform. Any person who may have attended criminal trials, where the plea of insanity is set up, knows the farce that is gone through with. He attended trials for weeks, when, on both sides, a large amount of testimony had been offered as to the insanity of the criminals, and when the trial had been concluded, the judge told the jury that twelve gentlemen had been called on either side, leaving it impossible for a jury to pass upon the question. Now, if a Commission should hear the testimony and then give its evidence, it is presumed it would have some influence with the court and jury. As it is now, it must be mortifying to all to see the way the subject is treated.

Another circumstance is, where Executive clemency is claimed or asked on the ground of insanity. Governor Morgan had asked for an appropriation for the purpose of a Commission. It is the course pursued by nearly all the governments of Europe. I am aware it would be attended with some expense; but, compared with the care and attention it would secure to the insane, this would amount to very little.

Dr. Wilbur favored the adoption of the resolutions, and related to the Society, briefly, accounts of his visits to some of the alms-houses of the State, showing the wretched condition of the insane poor of the State. Unless some Commission should be appointed, he could not see how the attention of the public could be called to the retrograde movement of removing insane patients from the lunatic asylums to the county poor-houses.

Dr. Coates said the only difficulty in the way was the manner of the appointment of this Commission. It would be well, if practicable, to empower this body to make such appointment. This would secure the appointment without prejudice or political influence.

Dr. Coventry said a petition on this subject had already been presented to the Senate, and referred to the Medical Committee of that body. The number of Commissioners, and the manner of their appointment, would be embodied in the bill, and the duty of framing it would devolve on that committee. He thought the less we meddle with legislation the better we should be off, excepting, however, to recommend measures conducive to the public health and good.

Dr. Parkhurst favored the adoption of the resolutions, and he deemed it necessary for the care and protection of the insane.

The question was then taken on the resolutions, and they were adopted.

The number of the committee was fixed at five, and the Chair appointed Drs. Coventry, Bradford, Coates, Mason, and Sanders such committee.

Dr. Squibb then read a communication from the Kings County Society, entitled "Notes upon New Remedies."

A motion to return the thanks of the Society to its author was unanimously adopted, and the paper referred to the Publication Committee.

The Secretary presented a communication from the Medical Society of Schuyler County. Referred to Publication Committee.

Dr. Ordronnaux next read a memorial from the Queens County Society, relative to the laws regulating the practice of physic and surgery. Accompanying it was a report made to the Queens County Society, on the subject, by Dr. John Ordronnaux, being a legal opinion. Referred to the Publication Committee.

Dr. Goodrich, from the committee appointed at the last meeting to inquire into the subject of "Anæsthetic Agency, its Origin, its Authorship, and its first Introduction into Medical and Surgical Practice in the United States," presented an elaborate report, and concluded by awarding to Dr. Wells the credit of being the discoverer of anæsthetic agency.

The report was accepted, and referred to the Publication Committee.

Dr. Staats moved the adoption of the report.

Dr. Griscom remarked, that long before Dr. Wells was born, Anæsthesia had been discovered, and that to Humphrey Davy belonged the credit for its discovery, if to any one. Dr. Wells did not discover the idea; he merely took it up and carried it forward. He claimed that Drs. Morton and Wells stood upon the same platform, and he was opposed to the Society saying that the claim of originality belonged to either of them.

Dr. Jones explained the connection of Drs. Wells, Morton, and Jackson with this subject, and thought the Society had better not have anything to do with the matter.

Dr. Bissell moved that the whole subject be laid on the table indefinitely. Adopted.

Dr. Parker, from the committee appointed at the last meeting to examine certain pharmaceutical preparations, presented their report, which was adopted, and referred to Publication Committee.

Dr. Percy read a paper on "Pharmaceutical Preparations," which was referred to the Publication Committee.

AFTERNOON SESSION.

The Society reconvened at 3½ o'clock.

Dr. Brinsmade, from the committee appointed to consider the recommendations of the American Medical Association on several subjects of importance, presented a report, accompanied by the following resolutions:

ON THE SUBJECT OF CRIMINAL ABORTIONS.

*Resolved,* That this Society cordially approves of the action of the American Medical Association in its efforts to exhibit the extent of the evils resulting from the procuring of criminal abortions, and of the means which are adopted to prevent its commission, and cheerfully comply with the request to a "zealous co-operation" for the furtherance of more stringent legislation, in regard to this most destructive and revolting crime, committed almost with impunity, and with appalling frequency.

*Resolved,* That a committee of three be appointed to present the memorial of the President and Secretaries of the American Medical Association, which has been read, to the Legislature of the State, at its present session.

The resolutions were adopted, and Drs. Staats, Armsby, and Townsend were appointed the committee.

ON THE NEW YORK STATE INEBRIATE ASYLUM.

*Whereas,* In the opinion of this Society, there is no hospital or asylum in our country so well calculated to relieve so much suffering and prevent so much insanity, idiocy, and death, as the New York

Inebriate Asylum, now in course of construction at Binghamton, where founded: Therefore,

*Resolved,* That this Society most earnestly recommend to the Legislature of the State of New York the importance of appropriating a sufficient sum of money for the immediate completion of the Inebriate Asylum.

*Resolved,* That a committee of three be appointed to present this action of the Society to the attention of the Legislature of the State, now in session, and to use their influence to obtain an enactment in accordance with the above resolution.

The resolutions were adopted, and Drs. Blatchford, March, and Quackenbush were appointed such committee.

Dr. Ordronnaux offered the following resolution, which was adopted:

*Resolved,* That a committee of five be appointed to report upon the feasibility of amending the present laws of this State regulating the practice of physic and surgery; and if so, in what way.

On this committee Drs. Ordronnaux, Jones, Mason, Willard, and Strew were appointed.

Dr. Coates offered a resolution that a committee of three be appointed to report upon the subjects presented in the Inaugural Address of the President, which was adopted, and Drs. Coates, Griscom, and E. H. Parker were appointed such committee.

Dr. Ball offered the following preamble and resolution:

*Whereas,* In view of the extensive adulteration of drugs which are sometimes sold by apothecaries, resulting often in great damage to the patient, and disappointment to the physician:

*Resolved,* That a committee of five be appointed by the Chair, of which Dr. Squibb shall be chairman, to report, at the next meeting of this Society, some measures calculated to correct this growing evil.

Adopted, and Drs. Squibb, Ball, Joel Foster, Percy, and Husted were appointed such committee.

Dr. Sanders then read a paper on "A Case of Insanity."

At the conclusion of its reading a motion was made to lay it on the table, which was carried.

A motion to reconsider the vote was lost.

Dr. Sanders then requested permission to withdraw the paper, which was granted.

Dr. William Taylor offered the following resolution:

*Resolved,* That a committee of three be appointed to present to the Legislature the subject of providing by law for a more general vaccination in this State, and that the report made by a committee to this Society, at its session of 1859, and the action of the Society thereupon, be communicated to the Legislature, with the request that a law be passed at its present session in conformity with the suggestions contained in said report.

Adopted, and Drs. William Taylor, Boyd, and Vanderpoel were appointed such committee.

The Secretary presented the following papers:

"A Case of Gun-shot Wound, the Ball passing through the Chest entire, and escaping from the Back," by N. C. Husted, of New York City.

"Facial Paralysis," by F. Everts, of Oswego.

Referred to Publication Committee.

The Society then adjourned until Thursday morning, at 9 o'clock.

THIRD DAY.

The Society was called to order at 9 o'clock, A. M.

The minutes were read and approved.

Dr. Parker, from the Committee on the President's Address, presented the following resolutions:

*Resolved*, That a committee of three be appointed by the President to represent this Society in the Medical Convention, for the revision of the Pharmacopœia, to be held at Washington, D. C., on the first Wednesday of May next, and that this committee be instructed to act in accordance with the recommendations of the President's Inaugural.

Adopted; and Drs. Squibb, Howard Townsend, and Caleb Green were appointed such committee.

*Resolved*, That a committee of five be appointed by the Chair to take into consideration so much of the President's Address as refers to a Topographical and Hydrographical Survey of the State, with reference to systematic drainage, as a hygienic measure, and to report at the next session of the Society.

Adopted; and Drs. Harris, Orton, Bradford, Seymour and Hunt were appointed such committee.

Dr. J. V. P. Quackenbush, Treasurer of the Society, made his annual report, which was referred to a committee composed of Drs. Mason, Beattie and Bradford.

Dr. Hoff presented a paper from Alfred Mercer, M.D., read before the Syracuse Medical Association, entitled "Prevention, Contagion, and Diagnosis of Small-Pox." Referred to Publication Committee.

Dr. A. Willard presented a biographical notice of Levi Farr, M.D., of Greene, Chenango County. Referred to Publication Committee.

Dr. Mason, from the committee to examine the Treasurer's books, reported that they had compared them with his vouchers, and that they find them correct. Report accepted.

Dr. Coventry, from the Committee on Nominations, presented the following report:

*President*—Daniel T. Jones, of Onondaga County.

*Vice-President*—E. H. Parker, Poughkeepsie.

*Secretary*—Sylvester D. Willard, Albany.

*Treasurer*—J. V. P. Quackenbush, Albany.

*Publication Committee*—Thomas Hun, S. D. Willard, and Howard Townsend.

The committee also reported upon the nominations to the various committees and delegates to the American Medical Association.

The committee recommended for the honorary degree of medicine the following persons: Francis J. D'Avignon, Clinton Co.; Harrison Teller, Brooklyn; Peter Moulton, New Rochelle.

The report of the committee was accepted, and the nominees, as presented by the committee, were elected to the respective positions for which they were named.

Dr. McNulty offered a resolution that the Society appoint delegates to attend the National Quarantine and Sanitary Convention, to be held at Boston, in June next. Adopted.

The Chair appointed twenty-five delegates, and by motion the name of the President was added to the list.

Dr. Foster called attention to a resolution, adopted at the last meeting of the Society, that County Medical Societies furnish the State Society with a complete list of the number of their members in each year, and of those who have died, together with the ages at which death took place.

Dr. Brinsmade presented the list of members, &c., of the Rensselaer County Medical Society. Referred.

Dr. Blatchford presented a condensed statement of what has been attempted in the direction of medical education, by the Medical Convention of 1846 and 1847, and by the American Medical Association since its organization in 1847. Referred to Publication Committee.

Dr. A. J. Dallas presented a biographical notice of Dr. Jas. Briggs, of Onondaga. Same reference.

Dr. Brinsmade presented the mortuary record of the City of Troy for nine years, from 1851 to 1859 inclusive; also a record of private practice for the years 1858 and 1859. Same reference.

Dr. Daniel Holmes, of Canton, Bradford Co., Penn., read a paper entitled "Fracture of the Neck of the Femur, within the Capsule, with Bony Union, in fourteen weeks and three days."

Dr. March offered the following resolutions:

*Resolved*, That we have listened with great interest to the paper just read by Dr. Holmes, on Inter-Capsular fracture of the neck of the thigh-bone; that the history of the accident, the symptoms, treatment and result, together with the examination of the post-mortem specimen, furnish satisfactory evidence of the existence of a fracture, as claimed by the author; that it was *complete*, not *impacted perfectly within the capsular ligament*, and so firmly united, as not to admit of separation without the use of great violence.

*Resolved*, That the thanks of the Society be presented to Dr. Holmes for his highly instructive and useful paper; and that he be requested to furnish a copy for publication in the Transactions of the Society.

Drs. Parker, McNulty and Brinsmade objected to the passage of the resolutions, for the reason that the specimen did not settle the disputed point as regards perfect bony union within the capsule, which could not be demonstrated in the specimen without a longitudinal section through the bone, and a careful microscopical examination.

Dr. Brinsmade then offered the following rosolution as an amendment:

*Resolved*, That the paper of Dr. Holmes, with the specimen of the bone, be referred to a committee of three, with Dr. March as Chairman, to report at the next meeting of the Society.

Adopted; and Drs. March, Brinsmade and E. H. Parker were appointed such committee.

Dr. French moved that a committee be appointed to address a letter to the Secretary of each County Medical Society here represented, requesting said Secretary to furnish the names of members of each County Society, and the names and age of all such members who have died for the last five years. Adopted, and Dr. French was appointed such committee.

Dr. Coventry offered the following resolutions, which were adopted:

*Resolved*, That the committee appointed to confer with the Medical Committee of the Legislature, on the subject of the appointment of a Commission of Lunacy, be discharged.

*Resolved*, That a committee of three, residing in the City of Albany, be appointed with authority, if such appointment cannot be effected at the present session of the Legislature, to present the subject early to the next Legislature.

Dr. Armstrong offered the following resolution:

*Resolved*, That the habit of prescribing, by regular physicians, articles of medicine, whether in the form of fluid extracts, sugar-coated pills, patent medicines, or other articles prepared by non-professional persons, or by persons ignorant of their therapeutical properties, or by persons not recognized by the medical profession as possessing the necessary qualifications, is incompatible with the honor, dignity and best interests of the profession, for the following reasons :

1st. Because the component parts of said medicines cannot be known with certainty.

2d. Because it is doing injustice to a useful class of persons, who, although not identified, are closely connected with the profession, and are justly regarded as auxiliary to its usefulness.

3d. Because it encourages a class of persons in no respect responsible for its honor and integrity.

4th. Because it commits the best interests of the profession to those who endeavor to profit by its sanction and patronage.

5th. Because it affords facilities and encouragement to non-professional persons, wholly incompetent to prescribe for themselves, and thus the profession is sometimes made to aid, by its sanction, the commission of criminal practice.

Adopted.

Resolutions were adopted returning thanks to the retiring officers, and also to the Mayor and Common Council of the City of Albany, for the use of the Common Council Chamber.

The Society then adjourned *sine die.*

